# Dr. John Arvid Ouchterlony

**Published:** 1905-11

**Authors:** 


Dr. John Arvid Ouchterlony, of Louisville, died at his home October 9, 1905, aged 67 years. He was born in Sweden where his early education was obtained; he came to the United States in 1857, began the study of medicine, and graduated from the University of the City of New York in 1861. In 1862 he entered the Union army as a medical officer, serving until 1865, and then located in Louisville. He taught medicine in the Louisville Medical College, in the Kentucky School of Medicine, and later became professor of medicine in the University of Louisville. He served as president of the Kentucky State Medical Society in 1890, as a vice-president of the American Medical Association at another time, and was a member of several other medical and scientific bodies. In 1891 King Oscar made him a knight of the Polar Star. Dr. Ouchterlony was a profound scholar, a genial companion, and a great teacher. Kentucky has suffered a great blow in his death and mourns her loss with the keenest grief.

